# The Impact of Policing on Involuntary Routes of Admission: A QIP on Patient Experiences

**DOI:** 10.1192/bjo.2022.306

**Published:** 2022-06-20

**Authors:** Mashal Iftikhar, Monika Gorny

**Affiliations:** 1 Barnet, Enfield and Haringey Mental Health NHS Trust, London, United Kingdom; 2 Camden and Islington NHS Foundation Trust, London, United Kingdom; 3 University College London, Division of Psychiatry, London, United Kingdom

## Abstract

**Aims:**

Patients often have negative experiences of police in their daily lives. Police involvement in mental health services can make an encounter feel disciplinary rather than therapeutic and exacerbate mental distress. People with mental illness, especially of minoritised backgrounds, are more likely to die after police contact, than other groups. Our aims were to: 1) explore patient experiences of being admitted onto the ward under section via the police, 2) explore patient understanding of the role the police play in mental health services 3) Use experiential data towards introducing trauma informed care in an inpatient setting

**Methods:**

A clinician administered questionnaire was conducted on an acute male inpatient ward, with 12 consenting male inpatient participants. All were involuntarily detained, ranging in age from 22 to 56 years; 11 out of 12 were of an ethnic minority background.

The questionnaire consisted of a mixture of open-ended questions and closed Likert scale questions with answers ranging from “strongly agree” to “strongly disagree”. Questions covered themes relating to the experience of admission and the ward environment, personal and communal experiences of policing, views on the role of policing in mental health service provision. Data were collated and presented in a local QIP showcase.

**Results:**

A significant split was identified between answers to open-ended and closed questions. When offering Likert based responses, 66% of participants felt safe with the police and believed that the police had a role in keeping people with mental health problems safe; 50% felt the police role should be greater in the future.

When responding to discussion-based questions, participants were critical of policing in relation to managing mental crises. Participants offered elucidative answers covering themes ranging from feeling a lack of agency, and the traumatic nature of criminalising mental distress, to concerns about abuse of power, the desire to limit the policing role to criminality and lack of trust engendered from experiences of racial injustice.

**Conclusion:**

Our results demonstrate that patient views on policing roles in mental health service provision are complex. The experiences of involuntary admission through the police are often traumatic, rooted in past police involvement in patient's lives. Although it is acknowledged that at times no feasible alternative is available in hostile situations, this QIP opened an important, previously avoided, discussion. This will hopefully lead to introduction of more trauma informed care in an inpatient setting.

